# Supplementary figures and images for: Towards a more general understanding of the algorithmic utility of recurrent connections
Source: PLoS Comput Biol. 2022 Jun 21;18(6):e1010227. doi: 10.1371/journal.pcbi.1010227 (PMC9258846; doi:10.1371/journal.pcbi.1010227)

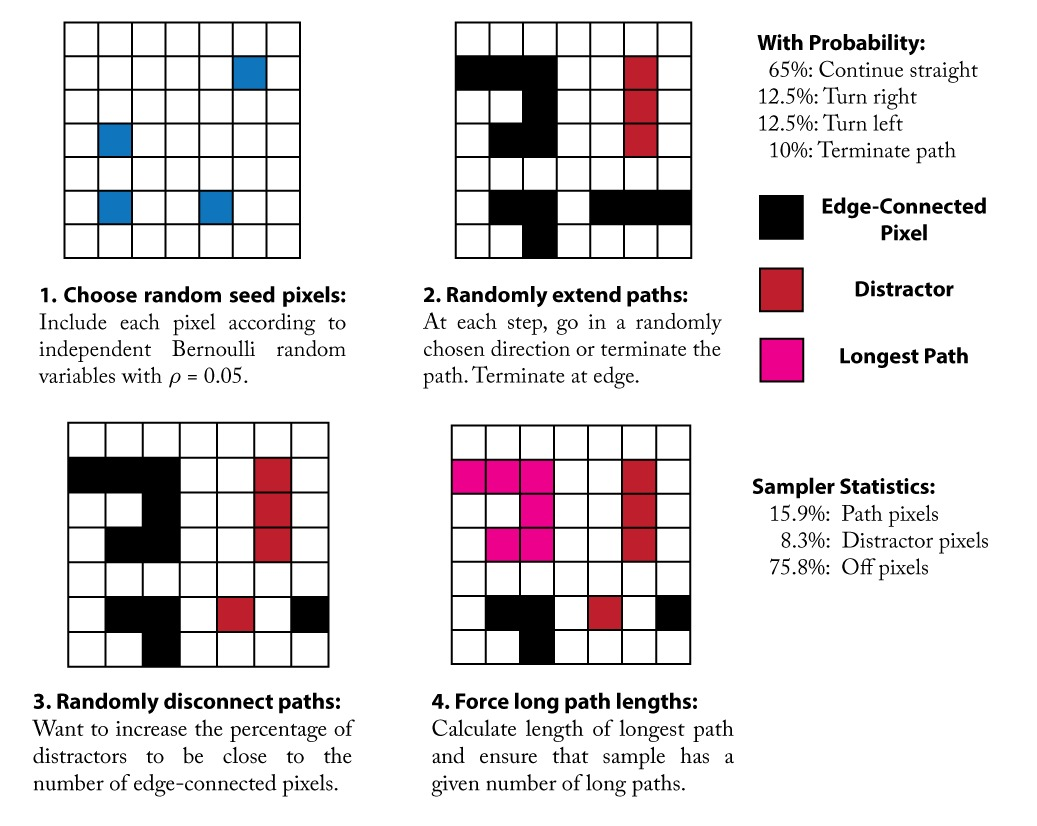

Supplement: S1 Fig — The procedure for randomly generating samples of the edge-connected pixel task with a specified distribution over paths lengths and a high percentage of distractors. When used to generate a set of 50,000 samples with path lengths evenly distributed between 1 and 25, the resulting sample has approximately 15.9% path pixels and 8.3% path pixels. (TIF) [file pcbi.1010227.s001.tif]

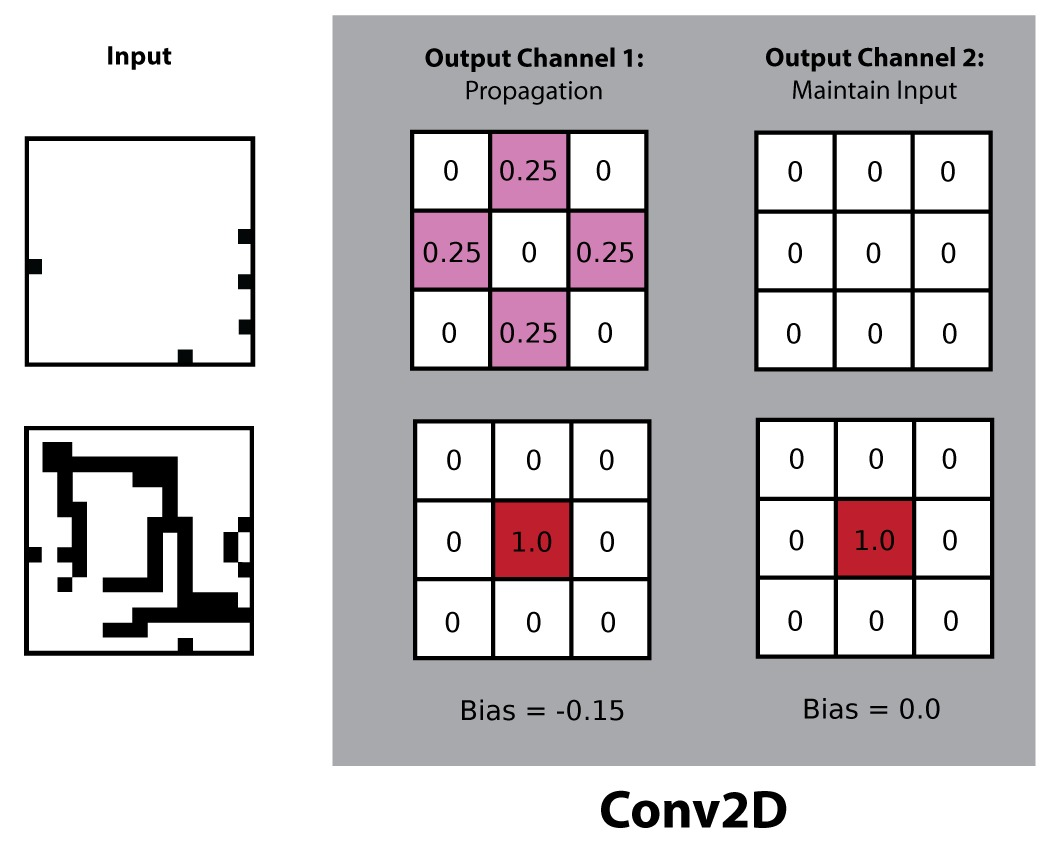

Supplement: S2 Fig — The tag propagation solution can be implemented via a sequence of repeated Conv2D layers with two input channels and two output channels. The two input channels are the current state of the tag propagation and the original input image. Output channel 1 performs one step of propagation while output channel 2 makes a copy of the original input image. (TIF) [file pcbi.1010227.s002.tif]

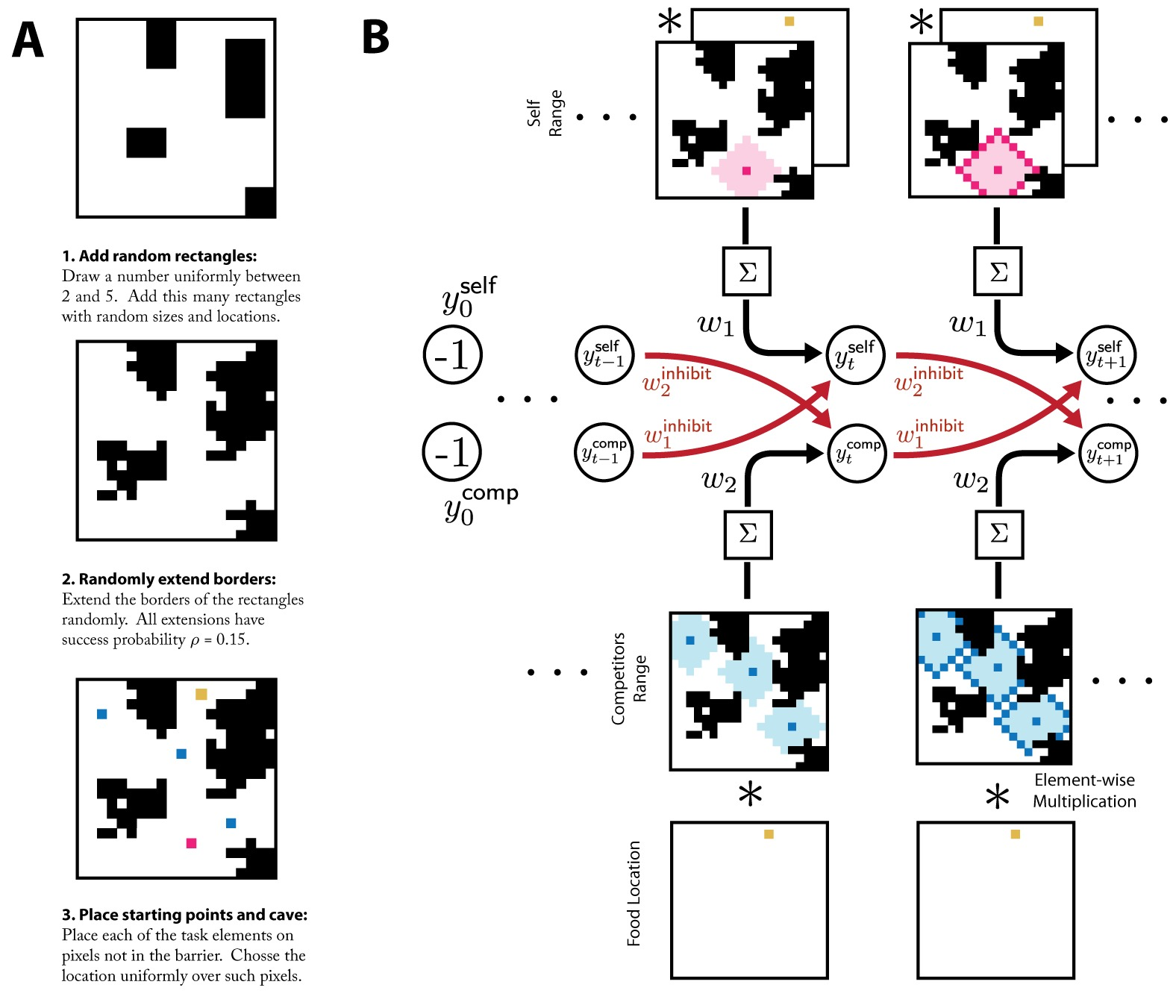

Supplement: S3 Fig — (A) Sample generation procedure for the competitive foraging task. (B) The recurrent decision network composed without output of the propagation network in the generalized tag propagation algorithm. At each time step the network extracts whether or not the food pixel is the in range of either group of animals. Once it comes into range for one group, the corresponding neuron activates and inhibits the other neuron. The active neuron in the final time step indicates which group was closer to the food. (TIF) [file pcbi.1010227.s003.tif]

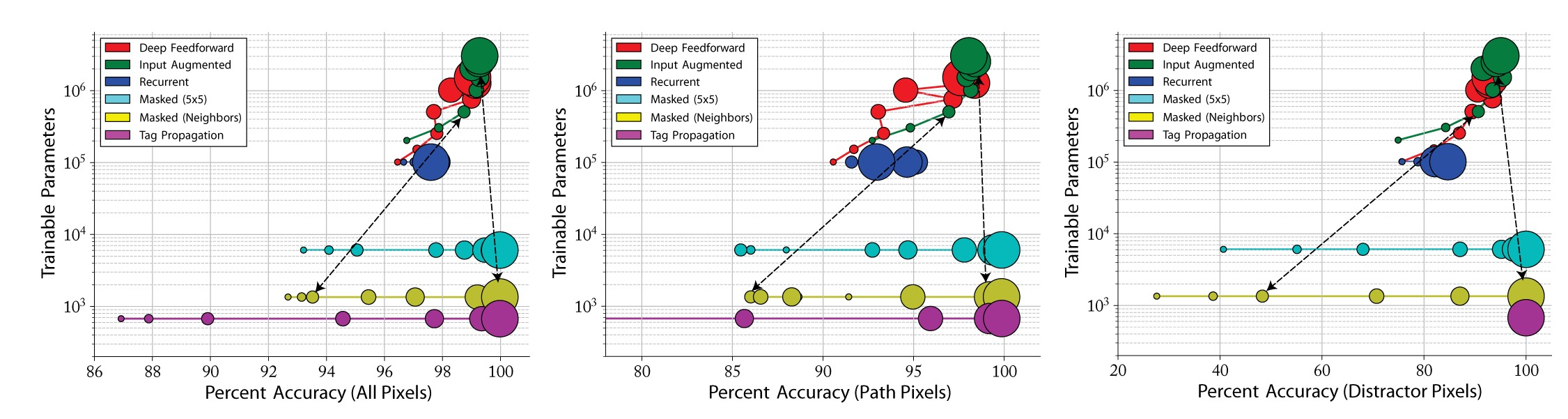

Supplement: S4 Fig — Performance of the best solution after hyperoptimization for each model type across layers with results sub-divided by error type. Models with weight sharing across layers have a constant number of trainable parameters as the number of layers is varied. The circle size indicates increasing layer number. Solid colored lines connect results for a single model type as the number of layers increases. Dotted lines with arrows highlight the masked and feedforward architectures with equivalent layers or timesteps for ease of comparison Here we plot the results for all 50 runs from random initializations for each layer and architecture combinations to give a sense of the variance across runs. These runs are post-hyperoptimization and thus all use the same set of optimal hyperparameters. S5 Fig shows the error on all pixels, S6 Fig on the path pixels, and S7 Fig on the distractor pixels. (TIF) [file pcbi.1010227.s004.tif]

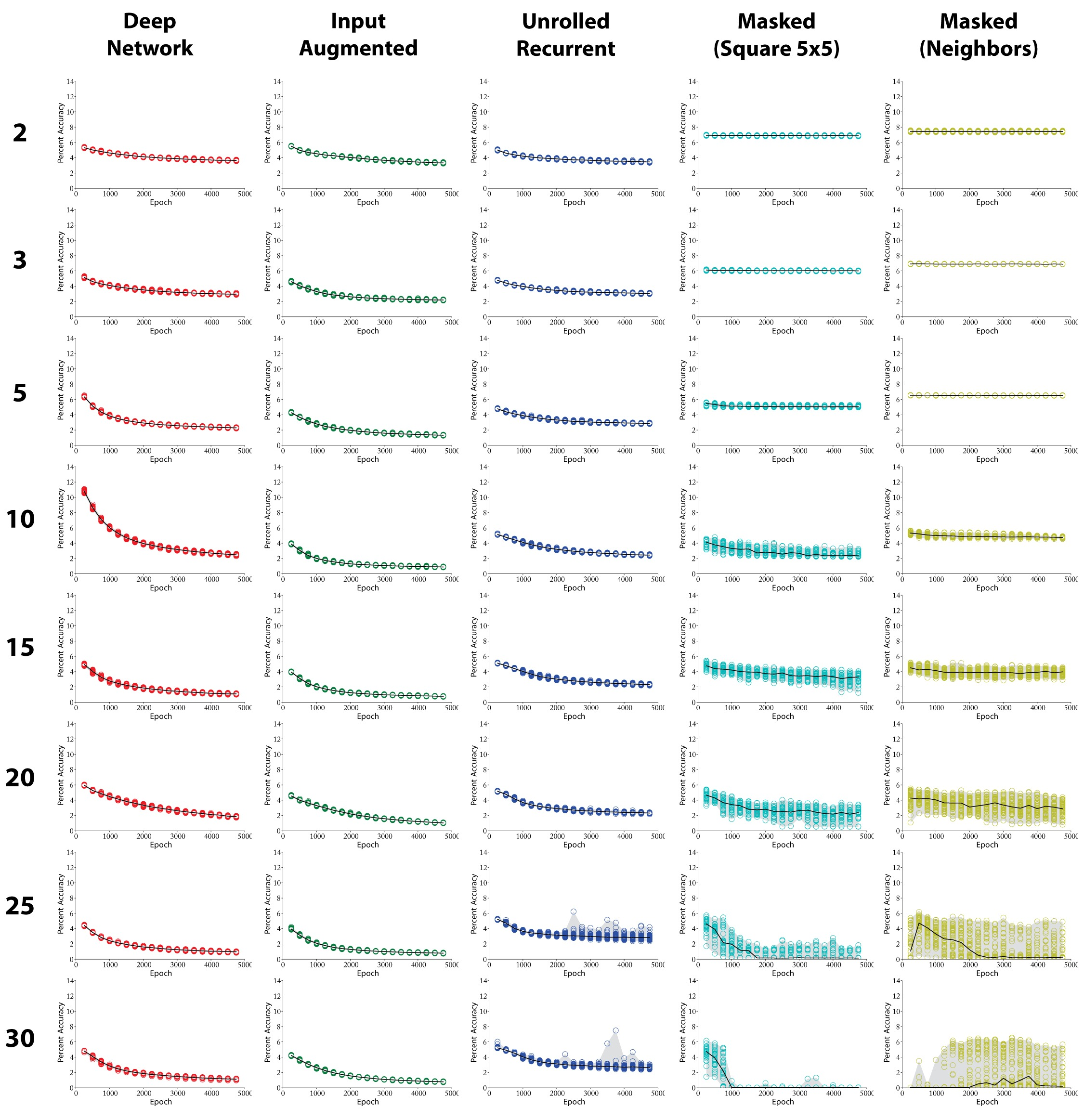

Supplement: S5 Fig — For each layer/architecture combination, 50 models were trained from random initializations for 50 epochs following hyperoptimization. Here we plot the error on all pixels every 250 epochs. The gray shading indicates the range of accuracies across all instantiations. (TIF) [file pcbi.1010227.s005.tif]

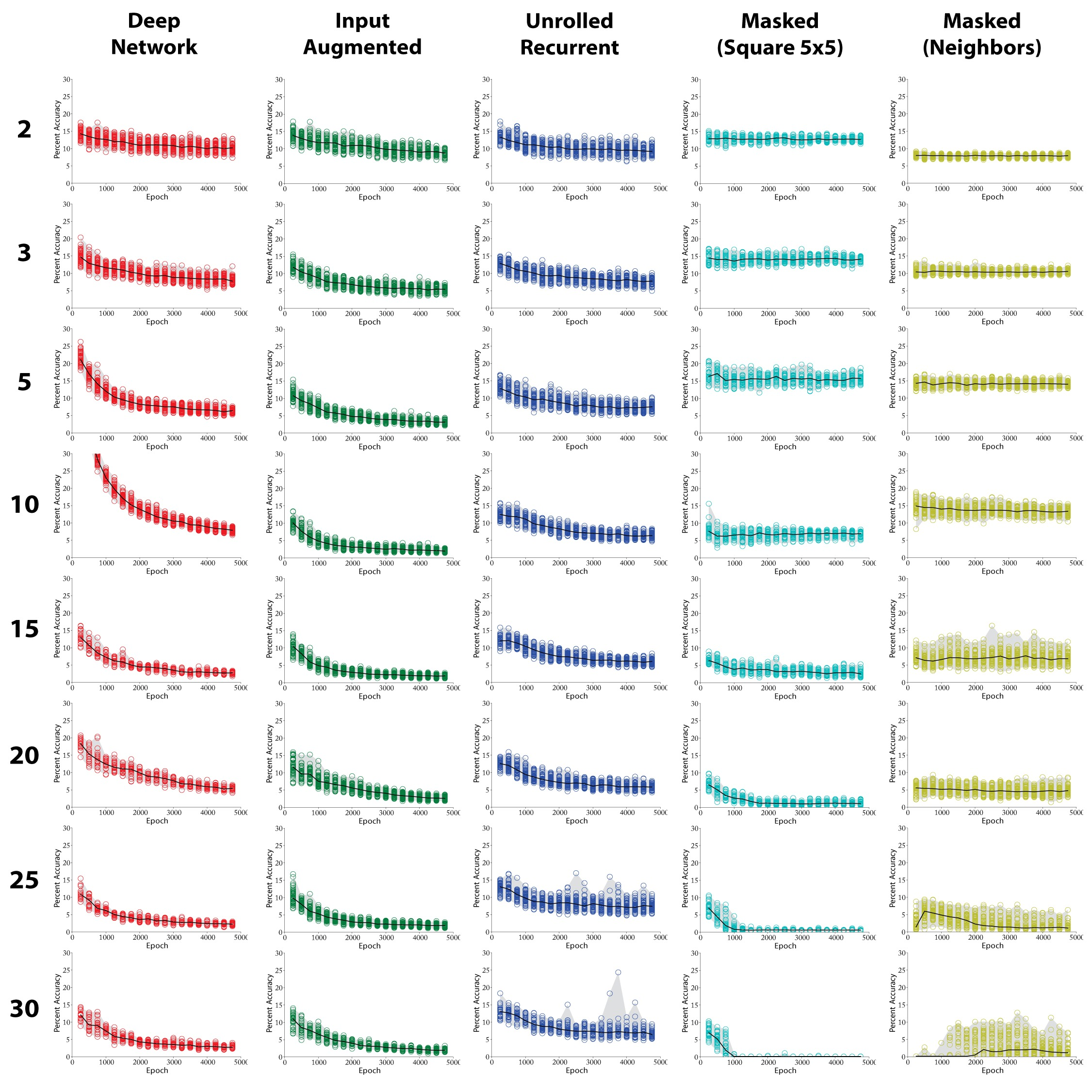

Supplement: S6 Fig — For each layer/architecture combination, 50 models were trained from random initializations for 50 epochs following hyperoptimization. Here we plot the error on path pixels every 250 epochs. The gray shading indicates the range of accuracies across all instantiations. (TIF) [file pcbi.1010227.s006.tif]

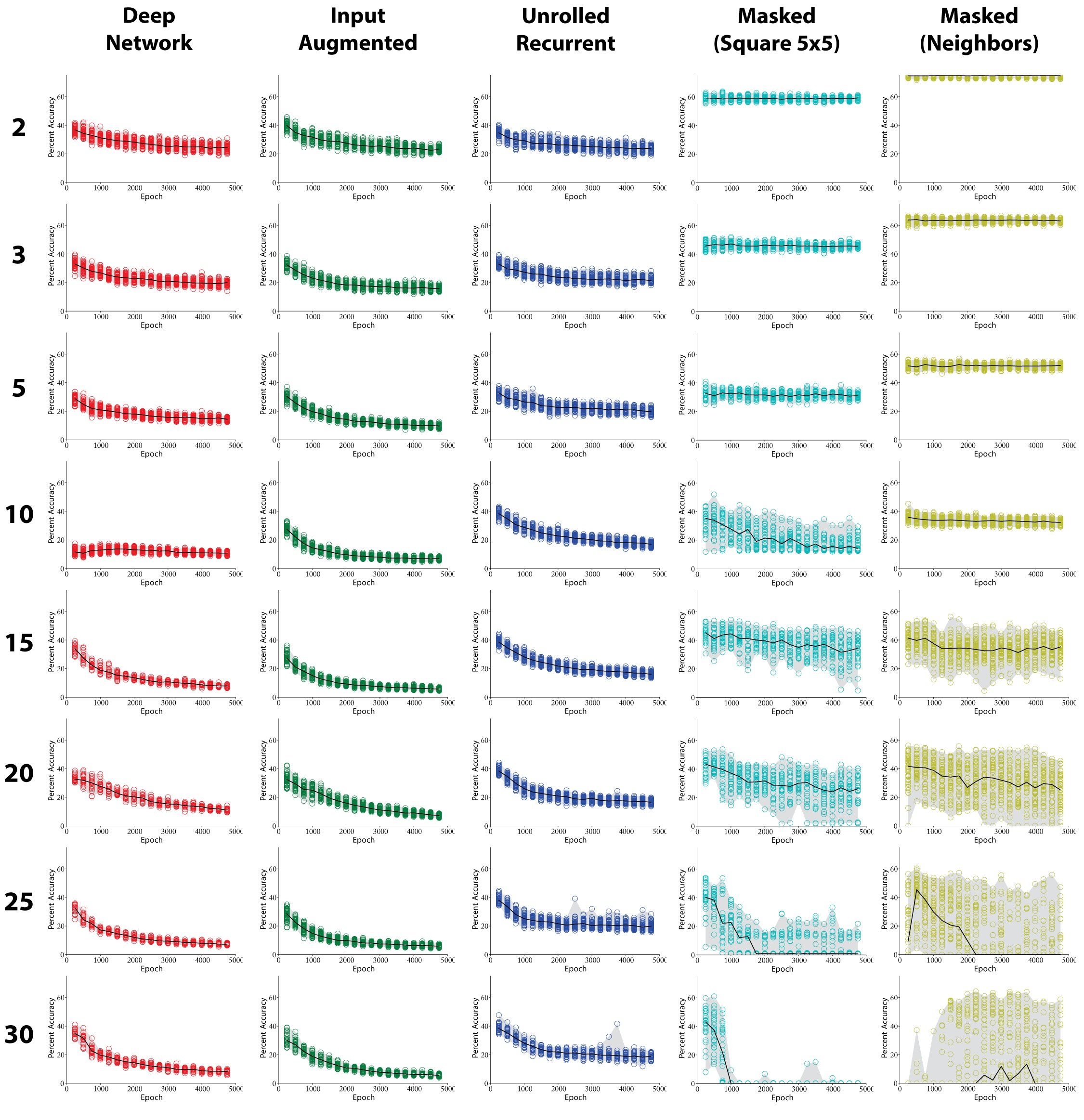

Supplement: S7 Fig — For each layer/architecture combination, 50 models were trained from random initializations for 50 epochs following hyperoptimization. Here we plot the error on distractor pixels every 250 epochs. The gray shading indicates the range of accuracies across all instantiations. (TIF) [file pcbi.1010227.s007.tif]
