# Supplementary material for: Towards a more general understanding of the algorithmic utility of recurrent connections
Source: PLoS Comput Biol. 2022 Jun 21;18(6):e1010227. doi: 10.1371/journal.pcbi.1010227 (PMC9258846; doi:10.1371/journal.pcbi.1010227)
Supplement: Attachment — Submitted filename: Response.pdf [file pcbi.1010227.s008.pdf]

Response to Reviewers

Summary of Major Changes

We thank the reviewers for their feedback which substantially improved the paper.

The primary changes to the study are additional comparisons to convolutional neural networks with multiple architectures for both tasks as suggested by the reviewers. For competitive foraging, the end-to-end task is a binary classification task and we added experiments training two different convolutional architectures on the task. For the edge-connected pixel task, we added experiments with the U-net architecture designed for image segmentation and clarified how tag propagation can be implemented through repeated convolutional layers.

In addition, we have expanded upon our results regarding hybrid networks. We have included new plots of per-pixel accuracy to better illustrate the intuition behind combining recurrent and feedforward networks and added results for a hybrid network which learns to perform per-pixel switching based on network performance on 10,000 samples.

Reviewer 1

In this paper, titled "Towards a More General Understanding of the Algorithmic Utility of Recurrent Connections", the authors explore various neural network architectures for their performance on an "edge-connected task". They find that feedforward networks provide a "quick and dirty" solution, that is outperformed by (some) recurrent architectures, when the latter are given enough time for the signal to propagate. I furthermore find the connection to food retrieval and path-length estimates very interesting. The paper is very well written, easily accessible, and the idea is interesting. I only have few smaller comments regarding technical aspects, as detailed below.

Thank you!

The larger issue that I have with this paper is that I am not entirely convinced that the simplistic, and somewhat narrow pixel-connection tasks are indeed suited to provide a more general understanding of the algorithmic utility of recurrence in biological systems (as claimed in the title and paper). What the paper does provide is a modern revisit of an interesting task in which recurrence is verified to be beneficial. However, I simply do not yet see how the results have broader implications beyond this simple use case (e.g. in real-world settings and in more natural tasks). Put perhaps too simplistic: what have we learned about the role of recurrence that we did not know before? Relatedly, I am uncertain in how far the results inform us about computations performed by the brain, as quantitative comparisons with neural data or human behaviour are currently missing.

I very much hope that my comments will be perceived as constructive as they are intended and that they may help the authors improve their manuscript going forward.

We thank the reviewer for sharing their frank opinion. We believe this comment is best answered in four parts.

First, briefly stated we feel that the main advance regarding the role of recurrence is a broader view of the non-temporal aspect of recurrent computation. Specifically, the ability to transform a complex, global decision into a local decision given the appropriate computation and propagation of a tag. While this intuition is present in earlier works, it is centered around the connection of connectivity itself and over time this intuition in our opinion has been somewhat submerged under the temporal use of recurrent in machine learning. Here we explicitly put the focus on the more general global-to-local transformation by tag propagation and demonstrate how by using multiple tags (not present in previous works) and more complex propagation and decisions on the propagation an even broader non-temporal set of computations can effectively utilize recurrent connections. We thus believe that this more robust description of non-temporal role of recurrence is a substantial advance in the understanding of the role of recurrence.

Second, we agree with the reviewer that our approach has been to tackle the question of the importance of recurrent connections from a more abstract perspective and that we have not included quantitative comparisons with neural data or human behavior. While comparisons with neural data or behavior would be valuable, we believe that our understanding of the role of recurrent computations requires advances at the computational and conceptual level before the appropriate experiments to extract relevant data become clear, hence the current manuscript is targeted at a computational journal. In the discussion we outline broad ideas for experiments related to our work on hybrid recurrent/feedforward networks inspired by (Kar and DiCarlo, 2021), but we also add that experimentally suppressing specifically recurrent components specifically is notoriously difficult.

Third, we certainly intend to explore more complex problems in the future, we see the current manuscript as an important step that is worth sharing with the community. Hopefully this study will inspire related work that will address more complex scenarios in the future by our group and others.

Fourth, we feel we have tried to be careful to not claim more than the contributions of the paper merit, both in qualifying the title with the terms "*toward*" and "*more* general", in the introduction, and in the discussion. We have now made further changes to the introduction and discussion emphasizing the abstract nature of the current work and how we intend it as a foundation for extensions into more real-world settings and neural data.

Major Comments

1. The hybrid approach feels very "engineered" based on the previous results, and I am not sure what we learn from the results given that the network architecture/behaviour is not a emergent in any one architecture. Relatedly, I am unsure in how far the results will generalise given that the hybrid solution feels "overfitted" to the task by combining separate elements that seemed to work well on the same task/dataset.

We completely agree that our specific implementation was engineered. We did not mean to claim this particular solution as a biologically relevant one. Rather, given the fact that circuits in the brain have both feedforward and recurrent components, and that most of the rest of the paper naturally focuses on the recurrent component, we wanted to demonstrate how even such a simple solution could already allow an interesting tradeoff.

We also point out that related ideas have been the core of other papers we reference such as (Spoerer et al., 2020) which argues that recurrent networks can provide a flexible trade-off between computational time and accuracy by varying the number of time steps used. Kar and DiCarlo have recently shown that one can make an image-per-image distinction between "early solvable" and "late solvable" images, with the interpretation that the late solved component is likely to rely on recurrent computations. Moreover, (Fyall et al. 2019) have shown that feedforward and feedback components interact to improve decodability of occluded images. We note that these papers all study different tasks than the image segmentation task we consider, including object recognition and shape discrimination, providing evidence that this network structure is of interest beyond this single task.

In addition, while we agree that our implementation of combining a recurrent and a feedforward solution may not be realistic as stated above, we do strongly feel that the utility of combining such solutions is not a result of overfitting but rather leveraging the strengths of the two different architectures. In general feedforward and recurrent architectures will generate different types of errors due to their different transformations and constraints. This is true broadly, but emphasized here specifically around the constraint that recurrent networks' greatest advantage, their ability to efficiently repeatedly apply transformations, also causes them to settle in a regime where they require many time steps to arrive at good solutions. If the different types of errors are systematic, as in our case when only a few time points are allowed to run, then circuits (learning) can make use of that fact to combine a recurrent and a feedforward architecture in a useful way. We felt this intuition is important to include due to the dual feedforward and recurrent nature of biological circuits. To further clarify the systematic different errors in different architecture component we both directly highlight by revising the relevant figure and perform more analyses where the switching is done per-pixel as different pixels show different patterns of errors depending primarily on their distance from the edge at low time steps.

All together, to better connect to this literature and emphasize the relevance of the computational experiments we rewrote that section of the results and extended the main relevant figure.

2. If I am not mistaken, the decision task was only trained on recurrent networks. Could the authors try feedforward networks on this task also?

We now added two feedforward CNNs trained on the competitive foraging task: a straightforward two-layer CNN and a ResNet-20. The task was setup for these networks as an end-to-end decision task such that the four binary images that define the task (environment, animal location, competitor location, food location) were inputted as a single image with four channels. ResNet-20 is less efficient than both the tag propagation network and the trained recurrent network in terms of the number of parameters; ResNet-20 has 269k trainable parameters while the tag propagation networks has 1354 parameters and

the trained recurrent network has 18.9k parameters. In our experiments, the ResNet-20 outperformed the trained recurrent decision network but still underperformed the tag propagation solution which achieves 0% error when run for a sufficient number of steps.

3. The task tested here is traditionally often solved by u-net feedforward structures (I here think of the edge-connection task as a way of image segmentation). Could the authors try this architecture on their data as well?

We thank the reviewer for the suggestion. During the revision we performed extensive experiments with U-net architectures. We have now added a section describing these architectures (and other convolutional architectures) to the results. In brief, we found that when using a similar number of neurons as the input augmented network, the U-net was not able to learn the edge-connected pixel task. However, if the number of neurons was greatly increased by doubling the number of channels in each layer, the U-net was able to achieve performance similar to the tag propagation algorithm and the masked networks. This most likely indicates that the inductive biases of translational invariance due to convolution and pass-through connections embodied in the U-net architecture greatly aids in learning the task in large networks; none of the other networks used in our experiments introduced this constraint. However, the recurrent networks we consider remain much more efficient in the number of neurons required to achieve highly accurate performance. We thank the reviewer for pointing us to explore these architectures.

Minor Comments

1. "This network architecture is illustrated in Figure 2B." Maybe Figure 2A is meant?

Yes, this has been changed to Figure 2A.

2. Figure 3's caption describes 4 panels (A-D), but only A-B are shown.

The extra captions C-D described subpanels that had been moved to other parts of the paper. Thank you for catching this, we have now deleted this reference.

References

1. Kar K, DiCarlo JJ. Fast recurrent processing via ventrolateral prefrontal cortex is needed by the primate ventral stream for robust core visual object recognition. Neuron. 2021;109(1):164-76. e5.
2. Spoerer CJ, Kietzmann TC, Mehrer J, Charest I, Kriegeskorte N. Recurrent neural networks can explain flexible trading of speed and accuracy in biological vision. PLOS Computational Biology. 2020; 16(10):e1008215.
3. Fyall AM, El-Shamayleh Y, Choi H, Shea-Brown E, Pasupathy A. Dynamic representation of partially occluded objects in primate prefrontal and visual cortex. Elife 2019

Reviewer 2

Summary: The authors seek to understand the relative effectiveness of feedforward neural networks, which have been wildly successfully in machine learning, compared to recurrent neural networks, which are more difficult to train for ML applications but much closer to the architecture of real neural circuitry. The two architectures, and hybrids of them, are tested on tasks that can exploit both local and nonlocal information: i) determining whether pixels in a visual image are connected using a tag propagation algorithm and ii) generalizing this approach to a minimal model for predicting future outcomes in a competitive foraging task. One of the highlighted results is that, while both feedforward networks and recurrent networks can learn to perform these tasks, which recurrent networks being able to perform it perfectly given enough time, constraints on computation can favor feedforward networks over recurrent networks. This is because the recurrent network solutions work inward from the starting pixels, and may not converge if the pixels being queried are too far apart. The feedforward networks, on the other hand, learn a different algorithm that does not rely on tag propagation and, although not perfect, outperforms tag propagation when computation time is limited. The authors show that a hybrid network architecture can learn to use the advantages of both approaches for a given amount of computation time.

Assessment: I enjoyed the paper and have only a few clarification/discussion points that I think would improve the presentation/understanding for the reader. I otherwise support publication in PLOS CB.

Best wishes,
Braden Brinkman

Main feedback

• Fig. 3: The caption mentions panels C and D, which do not appear or are not labeled in the figure.

The extra captions C-D described subpanels that had been moved to other parts of the paper. Thank you for catching this, we have now deleted this reference.

• P. 12: "This shows that the two network types excel in different computational regimes, the feedforward network at low computation time and the recurrent network at high computation time." I like this summary of the authors' result. Very clear.

Thank you!

• There are a few things I do not think I fully understand about Fig. 5 from the caption alone that could perhaps be clarified briefly in the caption.
--In panel B, what do the solid boundaries of the shaded diamond area represent in the last step shown? Just the boundary to where the agents can make it after a certain number of time-steps?

Panel B is meant to illustrator a single step of the propagation networks. The darker red pixels (and darker blue pixels for the competitor network) represent the new pixels added to the range by the most

recent propagation step. They will thus be any point that bordered the range at the previous step that is not part of the boundary. We thought it would be helpful to make this more salient and now include a description in the figure legend.

--Panel C: The red boundary of the yellow food pixel appears to make the pixel red unless the image is zoomed in. Perhaps remove the border? (Similarly in Fig. S2)

The red border has been removed and the color of these food pixels where changed to match the others. (All the food pixels were meant to be changed to a darker yellow without the red border; the ones pointed out by the reviewer were overlooked.)

--Panel D: This panel refers to the learning of the propagation of tags depicted in panel B, but it appears to show only the learned propagation of the competitors, is that correct? If so, noting that this is specifically the propagation of competitor tags and the animal tag propagation is not shown would be helpful.

We only show the competitors because the propagation network should be identical for both the competitors and the animal itself. All that changes is the input which are the starting location of the animals. We have added a note in the figure legend to clarify this.

--Panel D: It is also not clear to me what the red error pixels represent? Are these spots that would not be marked as blue (i.e., would remain white) by the network when they should be blue?

We view the output of the propagation network as a binary classification problem where each pixel should be labelled as in or out of the range (colored as blue and white respectively). The red pixels represent any misclassification. This can be either be a pixel is mistakenly included in the range (blue when it should be white) or mistakenly not included in the range (white when it should be blue).

--A legend to label the animal's decision in panel E as 'stay' or 'run' would be helpful to the reader. In the cases where both animal and competitors can reach the food, noting that the decision depends on whichever agent reaches the food first.

A label of "stay," "run," or "out of range" has been added to the examples in panel E. Here "out of range" means that in ten steps the food location was not accessible to either group of animals. A sentence has been added to the figure legend to clarify that the decision is based on which range included the food location first.

---This is explained clearly in the methods, but doesn't seem to be mentioned in the main text near Fig. 5. I think noting it in the main text would be useful as well (e.g., after "With these inputs each network was trained by stochastic gradient descent to output the correct shape of the tag at a given number of timesteps (see Methods for details)."

Thank you for the suggestion. We added a sentence afterwards to clarify: "In essence, the network should return all locations accessible to the animals in the specified number of time steps (see Methods for details on training)".

--I cannot parse the part of the sentence "the source pixels which being with the label"; is this a typo?

Yes this is a typo. The sentence has been modified to read "Unlike the edge-connected pixel task, the source pixels change in every sample to correspond to the location of the animal and its competitors respectively."

• While the competitive foraging task is of course a minimal model that does not capture a lot of complexity, it might be worth emphasizing when introduced on p. 14 that the animal has perfect knowledge of its environment (i.e., it knows with certainly the location of its competitors and the boundaries, which in a more realistic scenario may be unknown without some exploration).

We have added this point to our discussion of the task's limitations. The sentence now reads: "We note that this task does not of course capture all the complexities possible in judging the future utility of actions, and it assumes the animal has perfect knowledge of barrier and competitor locations which would require some exploration in most cases."

Minor comments and questions

• For the analytical solution for the pixel tag propagation network (eqs. 1-2), the network weights are assumed to be the same for all neurons due to homogeneity, and different equalities are assumed to hold for the edge/corner pixels. In principle, the distance from the edges seems like it could break the homogeneity between weights, rending inhomogeneous more optimal. Could this be an alternative possibility for why the trained networks do not learn a homogeneous solution (other than gradient methods not being guaranteed to find an optimal solution, as mentioned on p. 6)? I am just asking out of curiosity, I am not sure this is an important point to address in the paper, unless the answer is interesting and worth mentioning.

We share the same intuition that solutions using inhomogeneous weights are most advantageous in the case of low computational time, and likely this is the style of solution used by the networks we trained with low layer number. However, if we assume we have sufficient computational time, the same rule can govern how to update the state at every pixel which indicates we should have a solution with homogeneous weights (or more generally weights that perform the same function at each pixel possibly differing in the weights implementing the inequalities we described). This also lends an additional advantage which we now elaborate in the paper when we describe how the tag propagation can be implemented via repeated convolutional layers. Because the filter parameters are shared across all pixels, the number of trainable parameters will be independent of image size.

Regarding edge and corner pixels: We realized while adding the comparison to CNNs that a simpler way to describe the inequalities is to zero pad the image and then the same inequalities (eqs. 1-2) would hold for all pixels.

• P. 18: Recommend "Such networks instead of evolving hidden states include a component, dubbed an attention mechanism" -> "Instead of evolving hidden states, such networks include a component---dubbed an attention mechanism---"

Thank you. We have made this change.

• P. 21 (under eq. 6): "then this network is a support vector machine combined the kernel trick." -> "then this network is a support vector machine combined with the kernel trick." (?)

Thank you. Yes, the suggested change is correct and this sentence has been updated.

• For the competitive foraging model, the authors describe an exact/analytical solution in words in the Methods; is the solution expressible mathematically? e.g., do the weights in this case satisfy a system of inequalities like in eq. 2?

For the propagation networks, the same inequalities hold as we are performing tag propagation with different seed pixels. For the decision network in eq. 7, we want two things to be true: (1) w_1, w_2 should be large to make the sigmoid slope steep, i.e. $w_1 \geq 10$ and $w_2 \geq 10$, and (2) w_1^{inhibit}, w_2^{inhibit} should be much larger than w_1, w_2 such that they successfully inhibit their respective neuron, i.e. $w_1^{\text{inhibit}} > w_1$ and $w_2^{\text{inhibit}} > w_2$. For our implementation in the paper we used $w_1 = w_2 = 20$ and $w_1^{\text{inhibit}} = w_2^{\text{inhibit}} = 100$. This has now been added to the methods section.
